# Unpaid domestic work: persistence of gender-based labor division and mental disorders

**DOI:** 10.11606/s1518-8787.2023057004502

**Published:** 2023-05-30

**Authors:** Cíntia Maria Moraes Carneiro, Paloma de Sousa Pinho, Jules Ramon Brito Teixeira, Tânia Maria de Araújo

**Affiliations:** I Universidade Estadual de Feira de Santana Núcleo de Epidemiologia Feira de Santana BA Brasil Universidade Estadual de Feira de Santana. Núcleo de Epidemiologia. Feira de Santana, BA, Brasil; II Universidade Federal do Recôncavo da Bahia Núcleo Saúde, Educação e Trabalho Santo Antônio de Jesus BA Brasil Universidade Federal do Recôncavo da Bahia. Núcleo Saúde, Educação e Trabalho. Santo Antônio de Jesus, BA, Brasil

**Keywords:** Household Work, Home Environment, Mental Disorders, epidemiology, Gender and Health, Socioeconomic Factors

## Abstract

**OBJECTIVE:**

Evaluating characteristics of unpaid domestic work and its association with mental disorders, exploring gender differences.

**METHODS:**

We analyzed cross-sectional data from the second wave of an urban population cohort (n = 2,841) aged 15 and older from a medium-sized city in Bahia (BA). The representative population sample was randomly selected in subsequent multiple steps. We interviewed the survey participants at their homes. This study analyzed sociodemographic, occupational, unpaid domestic work and mental illness data, stratified by sex (gender). We investigated the association between the work-family-personal time conflict, the effort-reward imbalance in domestic and family work and the occurrence of common mental disorders, such as generalized anxiety disorder and depression. We estimated prevalence, prevalence ratios and their respective 95% confidence intervals.

**RESULTS:**

Among the participants, the unpaid domestic activities were performed by 71.3% of men and 95.2% of women, who were responsible for the investigated activities, except for minor repairs. The percentages of paid work were higher among men (68.1% versus 47.2% among women). The distribution of stressors and conflict experiences showed an inverse situation between genders: men depicted the highest high percentage of low work-family-personal time conflict (39.0%), while among women, the highest percentage was of high conflict (40.0%); 45.8% of the men reported low effort-reward imbalance in domestic and family work, while only 28.8% of women reported low imbalance. The investigated mental disorders were more prevalent among women, who showed a significant association between work-family-personal time conflict and common mental disorders, as well as depression; among men, conflict was positively associated with common mental disorders. The effort-reward imbalance, in turn, was strongly related to CMD (Common Mental Disorders), generalized anxiety disorder and depression among women. Amid men, this discrepancy was only associated to depression.

**CONCLUSIONS:**

Domestic work persists as a mostly feminine assigned activity. The stressful situations of unpaid domestic work and the work-family-personal time conflict were more strongly associated with adverse effects on the female mental health.

## INTRODUCTION

Unpaid domestic work has historically been neglected as a form of labor^1^. The determination of women’s suitability to the private/domestic space naturalized the idea that different unpaid activities carried out in this environment are female attributions and do not constitute a labor activity^2,3^.

The organizational structure of society and relationships in capitalism also determined gender social interactions^4^. From a complex system of power and social valuation, a gender-based division of labor was established following two criteria: the separation principle (masculine and feminine roles) and the hierarchical principle (masculine work is more valuable than feminine work)^3^. Linked to this structure, the conception that all activities which do not produce a commodity (value) are considered subaltern modalities was formed. This originated the bases that subjugate domestic work to the idea of an unproductive activity, which does not generate wealth and that is seen as inferior to production activities. Reproduction-oriented work was designated as a female assignment. Therefore, several characteristics were regarded as being naturally feminine, which submerges and erases historical social construction processes of valuation and devaluation systems; processes that supported gender-based labor division and hierarchical value attribution systems. These processes consolidated the invisibility and devaluation of domestic feminine labor, even when it became a paid job and was incorporated into the labor market^5^.

The context, experience and division of domestic work have historically been unequal between sexes (genders)^5^. Despite the emancipatory feminist movement and the female insertion in the labor market achieving significant advances, domestic activity, especially unpaid, remains a task that is not extensively shared with men^6^. Women remain primarily responsible for carrying out and keeping up unpaid domestic work. They are overloaded by having to reconcile their professional demands with home and family attention^10,11^.

In spite of the mechanisms that maintain the conditions of subordination and social invisibility, domestic work is crucial for human existence and is the basis for reproductive activities: society continues to exist because of its intrinsic care actions. In addition to generating well-being, this type of work consists of a set of daily activities that are crucial for human survival^12^. It is also carried out through actions aimed at planning, organizing and cleaning the house and direct personal care service actions directed to other individuals^13^. Consequently, it is theoretically possible to conceive domestic work as involving two dimensions: performing specific tasks (cleaning, cooking, washing, ironing, grocery shopping, among others) and caring activities (taking care of children, the elderly, sick and disabled people). These specific dimensions comprise varied levels of intensity, time investment and demand. Although they are not equivalent (both in effort quantity and intensity), the boundaries between these dimensions is tenuous since they can be performed concomitantly, due to the care involved in cooking, washing and ironing for others^13^. Nevertheless, two dimensions should compose the evaluation indicators of domestic work, allowing us to identify the workloads involved in these activities and the type of exposure to physical and mental health involved in their execution.

Domestic work, as well as other existing work activities, can be the source of mental health problems. Several factors contribute, directly and indirectly, to the well-being and health of people, such as the multiplicity of roles played, the high domestic workload^10^, the amount of time spent on household chores^14,15^, conflicts arising from the difficulties in reconciling different activities of work, family and time for health and leisure^16^, the lower perception of partner support when performing domestic work^17,18^, the unequal division of child care^9^ and house upkeep^19^, exposure to ongoing stress associated with housework^20^ and with stressful situations resulting from the imbalance between efforts and demands, and the low recognition of domestic work^21,22^.

At the international level, some available studies explore the relationship between unpaid domestic work and mental illness among men and women, whereas, in Brazil, this knowledge gap still persists. Predominantly, Brazilian studies are limited to focusing on the mental illness frequencies among women and their associated factors and, very rarely, focus on aspects related to domestic work^23^. A comparative analysis of the relationship between domestic work and health among men and women is also scarce.

Considering the aspects characterizing continuous processes of subordination and invisibility in reproductive activities described above, the role assigned to women in this occupation, coupled with the low research investment in Brazil on the analysis of this gender-based division of labor and the mental health of men and women, this study evaluated aspects of unpaid domestic work and its association with mental disorders, exploring gender differences. In the data exploration proposed here, two dimensions of domestic work were measured (both domestic and family care activities), as well as the occupational stressors present in this type of work and the work-family-personal time conflict., The analysis of these aspects allows us to identify that traditional models of gender-based labor division of unpaid domestic work persist, and their most relevant negative mental health outcomes among women.

## METHODS

We analyzed preliminary cross-sectional data from 2,841 participants in the second wave of the prospective cohort “Mental Health and Work Surveillance: a cohort of the population in Feira de Santana – BA”, funded by Fundação de Amparo à Pesquisa do Estado da Bahia (Bahia State Research Foundation, FAPESB)/Secretaria da Saúde do Estado da Bahia (Bahia State Health Department, SESAB), in partnership with the National Council for Scientific and Technological Development (CNPq) and Ministry of Health (MS), through the “SUS Research Program: shared management in health (PPSUS)” (PPSUS/BA – FAPESB/SESAB/CNPq/Ms 028/2018).

The cohort included individuals aged 15 or older, living in urban areas of the largest inland municipality in northeastern Brazil. The data derive from stratified sampling (sorted by sub district) with a two-stage cluster drawing: census tracts and streets (addresses). In each stratum (sub district), we performed a simple sampling of the primary sampling units (census tract). After that, we carried out a probabilistic sampling within each unit that was proportional to the number of households per street and estimated average number of people per household, divided by age group and sex, with data provided by the Brazilian Institute of Geography and Statistics (IBGE). From these estimates, we determined the number of streets that we should draw, which were later randomly selected. We included all households in the selected streets for interviews, in which residents aged 15 or older were eligible. In order to reduce losses, we conducted up to three home visits to interview each eligible resident. The visits happened on all days of the week, during the day, including Saturdays.

Data were collected between December 2018 and November 2019, through face-to-face interviews conducted in the participants’ own households. The interviewer was instructed to conduct the interviews, as far as possible, in order to favor the participants’ privacy, due to the subjective-focused interview questions. The research team was previously trained during workshops and meetings. We prepared a fieldwork manual of conducts and procedures to standardize data collection.

As data collection instruments, we used: a) household identification form, to record geographical location (sub district, census tract and address), register household residents (eligible or not) and housing conditions; b) multidimensional individual questionnaire, structured in ten theme blocks, from which we selected the data analyzed in this study (sociodemographic, occupational, domestic work and mental illness characteristics, stratified by sex).

In addition to the general characteristics of unpaid domestic work, we included the work-family-personal time conflict (CTFT) and effort-reward imbalance in domestic work (Domestic-DER).

The work-family-personal time conflict evaluates the impossibility or difficulty of reconciling roles assumed in the different life dimensions (work, family and personal time)^27^. The work-family conflict approach, based on an analysis by sex, articulates with the issue of gender-based labor division in the connection between paid work and domestic life as potential stress generators and, consequently, affecting the health of women and men inserted in professional work^16^. In this study, we built the indicator from the score sum of two questions that evaluated the conflict towards work interfering in the family and another one that evaluated the dimension of time for personal care and leisure. These items were answered on a Likert scale, with a zero to four score, validated for use in Brazil^16^. We used the total score to classify the work-family-personal time conflict as low, moderate or high, according to the tertiles.

The Domestic-DER scale assesses psychosocial stressors resulting from unpaid domestic and family work, based on the interaction of three dimensions: effort (extrinsic aspects, related to obligations and demands), reward (extrinsic aspects, related to recognition, work appreciation, and the feeling of justice) and excessive commitment (intrinsic component, related to personal involvement with their home and family assignments).^22^. This model’s hypothesis assumes that the imbalance between undertaken efforts and the rewards obtained with domestic and family work produces negative effects on health and life^22^. We evaluated this item using a scale that was translated and validated in Brazil^21^, containing 22 items with answers on a Likert-type scale (with a score of one to four). In this study, we composed the imbalance indicator using the sum of the scores of the dimensions: effort (seven items) and reward (ten items). The imbalance score was estimated by the equation: (Effort)/(Reward x correction factor), where values higher than one were subsequently classified into low, moderate and high Domestic-DER.

We evaluated mental disorders through widely used international research instruments which were validated in Brazil, showing good evaluation and measurement performance. Common Mental Disorders (CMD) were evaluated by a *Self-Reporting Questionnaire* (SRQ-20); Generalized Anxiety Disorder (GAD) was assessed by the anxiety module of the *Patient Health Questionnaire* (PHQ); and Depression was assessed by the PHQ-9.

SRQ-20 is a multidimensional instrument validated in Brazil^28^, consisting of 20 items with dichotomous response options (0-no and 1-yes), which evaluate symptoms of non-psychotic morbidity (insomnia, fatigue, irritability, forgetfulness, difficulty concentrating and somatic complaints). Suspicion of common mental disorders was defined by the selection of seven or more positive responses for women and five or more for men^29^.

PHQ^30^ evaluates mental disorders based on the criteria of the Diagnostic and Statistical Manual of Mental Disorders (DSM), cross-culturally adapted for use in Brazil^31^. The PHQ is organized into modules for measuring specific psychological disorders. For this study, we used the Generalized Anxiety Disorder (GAD) and depression modules. The GAD module contains seven items that assess how often the person has been bothered by anxiety-related problems in the past four weeks. The response options are expressed on a Likert scale, ranging from (0) “none” to (2) “more than half the days”. The presence of GAD is defined by four or more responses “more than half of the days” (2) and, necessarily, among them, a positive response to the item “feeling nervous, anxious, tense or very worried”^32^. We assessed depression using the PHQ-9, a validated version for use in Brazil^33^. This module evaluates the presence of symptoms in the last two weeks through nine items with four-point Likert response categories: never (0), less than a week (1), a week or more (2) and almost every day (3). We used the sum of the responses to calculate the total score, being ≥ 10 the cut-off point recommended to diagnose depression^32^.

This study sought to answer the following questions: does the difference in domestic work responsibility persist between genders? Are the work-family-personal time conflict and effort-reward imbalance in domestic and family work unevenly associated with mental disorders among men and women?

The analysis was exploratory and initially evaluated the characteristics of unpaid domestic work. Then, we estimated prevalence, prevalence ratio and respective 95% confidence intervals. We processed the analyses using the *Statistical software for data science* (Stata), version 15, and the *Statistical Package for the Social Science* (SPSS), version 24.

The study followed the recommended ethical criteria established for research involving humans and was approved by the Research Ethics Committee of the State University of Feira de Santana (CAAE: 74792617.4.0000.0053).

## RESULTS

The sample consisted of 2,841 people, mainly women (66.7%). For both sexes, the following characteristics predominated: age between 31 and 59 years old (men: 42.8%; women: 46.8%); not having a partner (men: 50.1%; women: 56.4%); high school as the schooling level (men: 47.2%; women: 45.5%); black people (men: 83%; women: 81%); monthly income up to one minimum wage (men: 47.2%; women: 59.5%). More than half of men had a paid job (68.1%), while only 47.2% of women were paid workers. Among them, informal jobs (men: 74.3%; women: 75.4%) and working hours of up to five days (men: 50.8%; women: 53.1%) predominated. Weekly workload of up to 30 hours was reported by 34.7% of men and 48.3% of women; full-time work (more than 30 hours per week) was reported by 65.3% of men and 56.2% of women (Table 1).

**Table 1:** Sociodemographic, occupational and unpaid domestic work characteristics, according to gender. Feira de Santana, Bahia, 2019, (n = 2,841).

Variables (n)	Men		Women		p
	
	n	%	n	%	
**Sociodemographic characteristics**					
Age range (years) (2,832)					
15–30	304	32.2	455	24.1	< 0.001
31–59	404	42.8	883	46.8	
≥ 60	236	25.0	550	29.1	
Marital status (2,796)					
With partner	461	49.9	816	43.6	0.002
Without partner	463	50.1	1056	56.4	
Schooling Level (2,796)					
Elementary school	371	40.1	718	38.4	0.060
High school	437	47.2	850	45.5	
Higher education	118	12.7	302	16.1	
Race/color (2,690)					
Non-black	150	17.0	343	19.0	0.216
Black	732	83.0	1465	81.0	
Monthly income (minimum wage^a^) (1,438)					
≤ 1	244	47.2	548	59.5	< 0.001
1–2	179	34.6	268	29.1	
> 2	94	18.2	105	11.4	
With whom they live (2,796)					
Family/friends	823	88.6	1709	91.5	0.012
Alone	106	11.4	158	8.5	
**Occupational characteristics**					
Paid work status (1,809)^b^					
Employed	448	68.1	543	47.2	< 0.001
Unemployed	210	31.9	608	52.8	
Working relationship (1,017)^c^					
Formal	117	25.7	138	24.6	0.698
Informal	339	74.3	423	75.4	
Working days (per week) (959)					
≤ 5	222	50.8	277	53.1	0.485
6–7	215	49.2	245	46.9	
Working hours (per week) (927)					
≤ 30	144	34.7	224	43.8	0.005
> 30	271	65.3	288	56.2	
**Housework**					
Does domestic activity (or activities) (s) (n = 2,483)					
No	217	28.7	83	4.8	< 0.001
Yes	539	71.3	1644	95.2	
Doing the laundry (2,631)					
No	471	58.8	283	15.5	< 0.001
Yes	330	41.2	1547	84.5	
Ironing (2,618)					
No	633	79.1	884	48.6	< 0.001
Yes	167	20.9	934	51.4	
Cleaning the House (2,637)					
No	325	40.5	153	8.3	< 0.001
Yes	478	59.5	1681	91.7	
Cooking (2,632)					
No	447	55.9	236	12.9	< 0.001
Yes	353	44.1	1596	87.1	
Grocery/supermarket shopping (2,625)					
No	333	41.7	391	21.4	< 0.001
Yes	465	58.3	1436	78.6	
Making small repairs (2,619)					
No	361	45.1	1290	70.9	< 0.001
Yes	439	54.9	529	29.1	
Taking care of children ≤ 5 years old (2,582)					
No	673	86.2	1269	70.5	< 0.001
Yes	108	13.8	532	29.5	
Taking care of the elderly, sick people or with special needs (2,588)					
No	710	90.6	1478	81.9	< 0.001
Yes	74	9.4	326	18.1	
Receives support in domestic work^d^ (2,406)					
Yes	322	48.8	864	49.5	0.760
No	338	51.2	882	50.5	
Hired a housekeeper (2,760)					
Yes	67	7.3	109	5.9	0.149
No	847	92.7	1737	94.1	
CTFT^e^ (1,038)					
Low	139	39.0	196	28.8	0.003
Moderate	97	27.3	213	31.2	
High	120	33.7	273	40.0	
Domestic-DER^e^ (2,071)					
Low	228	45.8	447	28.4	< 0.001
Moderate	102	20.5	588	37.4	
High	168	33.7	538	34.2	

^a^ Minimum wage in 2019: R$ 998.00.

^b^ We considered the participant’s current working condition, excluding students, retirees, pensioners, housewives or those who lived on income.

^c^ This N considered work bonds in the last year (regardless of current job).

^d^ Support in the performance of domestic activities, according to who provided it, had the following distribution (possibility of multiple responses): domestic worker: 5.8%; other men: 18.8%; husband/partner: 23.1%; wife/partner: 21.7%; other women 89.4%.

^and^ CTFT (work-family-personal time conflict) was answered by people who had paid work in the last year and did domestic activities and Domestic-DER (effort-reward-domestic and family imbalance) was answered by people who said they performed unpaid domestic work.

The survey pointed out that women are mainly responsible for carrying out domestic activities, assumed by 95.2% of the female participants, while 71.3% of men performed this type of task. This picture changes when we observe the data of minor repairs that were reported by 54.9% of men and 29.1% of women. Among the household activities which women were the most responsible for, we found: doing the laundry (men: 71.3%; women: 95.2%); ironing clothes (men: 20.9%; women: 51.4%); cleaning the house (men: 59.5%; women: 91.7%); cooking (men: 44.1%; women: 87.1%); grocery shopping (men: 58.3%; women: 78.6%).

The attribution of domestic work involving family care activities wase also assumed mainly by women, among them: caring for children up to five years old (men: 13.8%; women: 29.5%) and caring for the elderly, sick or people with special needs (men: 13.8%; women: 29.5%). Having support to perform domestic work was reported by 51.2% of men and 50.5% of women. We also evaluated who offered the support – housekeeper, spouse, other women (mother/sister/daughter/neighbor) or other men (father/brother/son/neighbor): support received from another woman had the highest percentage (89.4%). Support from a housekeeper was reported by 7.3% of men and 5.9% of women.

Among women, a situation of high conflict work-family-personal time predominated (40.0%), followed by moderate conflict (28.8%); among men, the highest percentage was low conflict (39.0%). For the effort-reward housework imbalance, moderate level predominated among women (37.4%), followed by high level (34.2%); among men, the low level prevailed (45.8%) (Table 1).

In the study population, the prevalence of mental disorders was more significant among women: the prevalence of common mental disorders was 25.5% against 21.7% of men; of generalized anxiety disorder was 6.8% among women and 2.4% among men; and depression was found in 10.9% of women and 5.5% of men (Data not presented in tables).

The high level of work-family-personal time conflict was significantly associated with common mental disorders in both men (PR = 1.69; 95%CI: 1.12–2.55) and women (PR = 1.86; 95%CI: 1.31–2.66), and depression only among women (PR = 1.99; 95%CI: 1.05–3.75) (Table 2).

**Table 2:** Prevalence (%), prevalence ratios and 95% confidence intervals for the association of mental disorders, according to levels of effort-reward imbalance and work-family conflict and according to gender. Feira de Santana, Bahia, 2019.

Variables	Men				Women			
	
	P	95%CI	PR	95%CI	P	95%CI	PR	95%CI
**Common Mental Disorders**^a^								
Work-family conflict								
Low	20.9	14,4–28,8	1.00	–	17.3	12.2–23.4	1.00	–
Moderate	24.0	15.8–33.7	1.15	0.71–1.86	24.6	18.9–31.0	1.43	0.97–2.11
High	35.3	26.7–44.8	1.69	1.12–2.55	32.2	26.6–38.2	1.86	1.31–2.66
Domestic-DER								
Low	24.7	19.1–30.9	1.00	–	16.7	13.3–20.5	1.00	–
Moderate	34.0	24.8–44.1	1.38	0.97–1.97	26.3	22.7–30.1	1.57	1.23–2.02
High	28.4	21.5–36.0	1.15	0.82–1.61	30.6	26.6–34.7	1.83	1.43–2.34
**Generalized Anxiety Disorder**^b^								
Work-family conflict								
Low	1.4	0.2–5.1	1.00	–	4.7	2.1–8.6	1.00	–
Moderate	3.1	0.6–8.8	2.13	0.36–12.6	6.2	3.3–10.3	1.32	0.58–3.02
High	3.4	0.9–8.4	2.32	0.43–12.5	8.2	5.2–12.2	1.76	0.83–3.74
Domestic-DER								
Low	2.2	0.7–5.1	1.00	–	3.4	1.9–5.6	1.00	–
Moderate	2.0	0.2–6.9	0.89	0.17–4.46	7.8	5.7–10.3	2.30	1.30–4.06
High	5.4	2.5–10.0	2.42	0.83–7.12	8.3	6.1–11.0	2.44	1.37–4.32
**Depression**^c^								
Work-family conflict								
Low	3.6	1.1–8.3	1.00	–	6.2	3.2–10.6	1.00	–
Moderate	3.1	0.6–8.7	0.85	0.21–3.47	11.9	7.8–17.0	1.92	0.99–3.71
High	9.3	4.7–16.1	2.55	0.91–7.15	12.4	8.6–16.9	1.99	1.05–3.75
Domestic-DER								
Low	4.8	2.4–8.4	1.00	–	4.7	3.0–7.2	1.00	–
Moderate	7.8	3.5–14.9	1.63	0.67–3.92	10.7	8.3–13.5	2.26	1.40–3.65
High	10.8	6.5–16.5	2.23	1.08–4.61	13.9	11.1–13.9	2.93	1.83–4.67

P: prevalence; PR: prevalence ratio.

^a^ Assessed by SRQ-20.

^b^ Assessed by the PHQ anxiety module.

^c^ Assessed by PHQ9.

Among women, moderate and high levels of effort-reward imbalance were significantly associated with common mental disorders (PR = 1.57; 95%CI: 1.23–2.02; and PR = 1.83; 95%CI: 1.43–2.34, respectively), generalized anxiety disorder (PR = 2.30; 95%CI: 1.30–4.06; and PR = 2.44; 95%CI: 1.37–4.32, respectively), and depression (PR = 2.26; 95%CI: 1.40–3.65; and PR = 2.93; 95%CI: 1.83–4.67, respectively). Among men, high Domestic-DER was significantly associated only with depression (PR = 2.23, 95%CI: 1.08–4.61).

## DISCUSSION

The results of this study demonstrated the persistence of gender-based labor division, both in aspects related to paid and unpaid activities, with greater disadvantages for women. The proportions of exclusion from the labor market, lower monthly income, lower wages, informal paid work, and part-time work were also higher among women than the proportions observed among men. These aspects, which show gender differences in participation in the labor market and access to its benefits, may influence the increase in the time and intensity of participation in unpaid domestic and care activities^5,8^. The lower access to income hinders professional support when performing these activities (whether through daycare centers, hiring a paid housekeeper). At the same time, lower participation in the labor market favors higher responsibility for domestic work, which, in turn, limits the possibilities for qualification and vocational training. Thus, the historically higher responsibility handicaps the access of women to the labor market^7^, promoting and fostering the exclusion cycles and perpetuating the mechanisms of production and reproduction of gender inequalities.

Most of the participants reported domestic activities. However, women were the main responsible for these activities, except for small repairs. Previous studies identified little masculine participation in domestic activities, classifying it as occasional, flexible and limited to small repairs^1^. Activities such as taking care of children, cleaning, doing the laundry, tidying up and cooking are still mostly performed by women^10^. Therefore, we observed that the patriarchal structuring conceptions that assign women the responsibility of unpaid domestic activities, regardless of their insertion in the labor market, continue.

This study showed a significant impairment of female mental health, corroborating with the existing literature, which points to a higher prevalence of mental disorders among women. The most frequent disorders were those related to anxiety symptoms, depressive mood, insomnia, anorexia nervosa and psychophysiological symptoms^10^. Meanwhile, among men, conduct disorders stand out, such as drug use and alcohol abuse^10^.

International studies consistently showed an association between unpaid domestic work characteristics and physical and mental health^9,14,15,17^. Brazilian studies also evidenced higher rates of mental illnesses among women^10,21^. When we evaluated both sexes, the higher negative effect on female mental health became evident^16^, similar to international studies.

The data reveal that women were not only more involved and primarily responsible for domestic work, but also experienced higher conflict percentages in the different dimensions of life (work-family-personal time) and were more affected by the domestic work stressors, facing situations of effort-reward imbalance. Consequently, it is harder/impossible for women to reconcile multiple roles. The associations between these aspects and mental illness were consistent for women, suggesting that differences in the performance of unpaid domestic work are related to the most prominent adverse effects among them.

A study that evaluated the association between work-family-personal time conflict and generalized anxiety identified a higher conflict and prevalence proportion of anxiety disorder in women, as well as a stronger association between the variables^16^.

Exposure to the effort-reward imbalance negatively affected mental health. The devaluation of domestic activities, the higher responsibility, the volume of performed tasks, as well as the feeling of injustice during work execution are factors that unbalance the relationship between efforts necessary to develop such activities and the granted rewards/recognition, generating stressors that are associated with physical and mental illness, especially for women^21^.

This is an exploratory study, aimed at analyzing characteristics of unpaid domestic work, focusing on its distribution and evaluating its relationship with the most frequent mental disorders in urban populations (common mental disorders, depression and generalized anxiety disorders). Hence, the obtained results should be analyzed cautiously, since they correspond to an initial approximation of analyzes between these events, without considering adjustments or interactions between variables. Despite this limitation, we investigated a representative sample from the target population of the largest countryside city in northeastern Brazil, including a sample of men and women. Additionally, we analyzed a reproduction-related life dimension, which receives little attention. When analyzed, it is generally restricted to the analysis of women.

The evaluation of stressors in domestic work in a Brazilian context, from a sample of the general population, used a formerly restricted powerful data production tool. The analysis in this direction showed the persistence of disadvantages for women not only in the assignment of responsibilities for the home and family, but also identified the production of stressors associated with these assignments. The instrument (Domestic-DER), adapted from one of the most widely used occupational stressors models in the paid work world^34^, brings new possibilities to better identify exposures in this type of work, still hidden and devalued. Thus, it allows us to glimpse the analysis of useful indicators to identify unfavorable situations, which can guide actions, public policies and alternatives to reduce social gender-related inequalities in health.

We observed persistent inequalities in the division of unpaid domestic work according to gender and greater repercussions on female mental health. It is likely that the covid-19 pandemic has intensified these inequalities, since the support network (schools, kindergartens, relatives, babysitters) became unavailable and the demands for cleaning expanded in this context. Moreover, remote work brought paid activities home, accumulating, in the same space, the world of work and family care without clear time boundaries. The overlap of requirements and demands at home can leverage conflicts and occupational stressors. The discussion of the relationship between gender/domestic work/mental health remains hidden, but the results obtained here showed important inequality indicators that need to be addressed as public health problems. It is necessary to break down the walls that enclosed domestic work in the private sphere and analyze it as an illness determinant. Pre-and post-pandemic comparative studies can scale new consequences of this equation and contribute to the elaboration of public policies to reduce the identified inequalities.
