# Supplementary material for: Unpaid domestic work: persistence of gender-based labor division and mental disorders
Source: Rev Saude Publica. 2023 May 30;57:31. doi: 10.11606/s1518-8787.2023057004502 (PMC10231910; doi:10.11606/s1518-8787.2023057004502)
Supplement: Translation

# Translation: Trabalho doméstico não remunerado: persistência da divisão sexual e transtornos mentais

**Authors:** Cíntia Maria Moraes Carneiro, Paloma de Sousa Pinho, Jules Ramon Brito Teixeira, Tânia Maria de Araújo

**Affiliations:** I Brasil Universidade Estadual de Feira de Santana. Núcleo de Epidemiologia. Feira de Santana, BA, Brasil; II Brasil Universidade Federal do Recôncavo da Bahia. Núcleo Saúde, Educação e Trabalho. Santo Antônio de Jesus, BA, Brasil

**Keywords:** Trabalho Doméstico, Ambiente Domiciliar, Transtornos Mentais, epidemiologia, Saúde de Gênero, Fatores Socioeconômicos

## Abstract

**OBJETIVO:**

Avaliar características do trabalho doméstico não remunerado e sua associação com transtornos mentais, explorando diferenciais de gênero.

**MÉTODOS:**

Neste estudo foram analisados dados transversais da segunda onda de uma coorte da população urbana (n = 2.841) com idade a partir dos 15 anos de uma cidade de médio porte da Bahia (BA). A amostra representativa da população foi aleatoriamente selecionada em etapas múltiplas subsequentes. As entrevistas foram realizadas nos domicílios dos participantes do levantamento. O estudo analisou dados sociodemográficos, ocupacionais, do trabalho doméstico não remunerado e adoecimento mental, estratificadas por sexo. Investigou-se associação entre o conflito trabalho-família-tempo para si, o desequilíbrio esforço-recompensa no trabalho doméstico e familiar e a ocorrência de transtornos mentais comuns, de transtorno de ansiedade generalizada e de depressão. Foram estimadas prevalências, razões de prevalência e respectivos intervalos de confiança de 95%.

**RESULTADOS:**

Entre os participantes, verificou-se que as atividades domésticas não remuneradas eram realizadas por 71,3% dos homens e 95,2% das mulheres, que se mostraram as principais responsáveis pelas atividades de trabalho investigadas, exceto pequenos consertos. A inserção em trabalho remunerado foi maior entre os homens (68,1% contra 47,2% entre as mulheres). A distribuição dos estressores e experiência de conflitos evidenciou situação inversa entre homens e mulheres: o maior percentual entre os homens foi de baixo conflito trabalho-família-tempo para si (39,0%), já entre as mulheres, maior percentual foi de alto conflito (40,0%); entre os homens, 45,8% referiram baixo desequilíbrio esforço-recompensa no trabalho doméstico e familiar, enquanto apenas 28,8% das mulheres relataram baixo desequilíbrio. Os transtornos mentais investigados foram mais prevalentes entre as mulheres, que apresentaram significativa associação entre o conflito trabalho-família-tempo pessoal e os transtornos mentais comuns e a depressão; entre os homens o alto conflito foi associado aos transtornos mentais comuns. Já o desequilíbrio esforço-recompensa se mostrou fortemente relacionado aos TMC, ao transtorno de ansiedade generalizada e à depressão entre as mulheres. Entre os homens, esse desequilíbrio relacionou-se apenas à depressão.

**CONCLUSÕES:**

O trabalho doméstico persiste como atribuição majoritariamente feminina. As situações estressoras do trabalho doméstico não remunerado e o conflito trabalho-família-tempo para si associaram-se mais fortemente aos efeitos adversos na saúde mental das mulheres.

## INTRODUÇÃO

O trabalho doméstico não remunerado tem sido historicamente negligenciado como forma de trabalho^1^. A determinação de adequação das mulheres ao espaço privado/doméstico naturalizou a ideia de que as diferentes atividades realizadas nesse ambiente, de forma gratuita, são atribuições femininas e não constituem uma atividade laboral^2,3^.

A estrutura de organização da sociedade e das relações no capitalismo também determina as relações sociais de gênero^4^. A partir de um complexo sistema de poder e de valorização social estabelece-se uma divisão sexual do trabalho estruturada em dois princípios: o da separação (o trabalho dos homens e o das mulheres) e o princípio hierárquico (o trabalho do homem é mais valioso do que o da mulher)^3^. Atrelada a essa estrutura, desenvolve-se a concepção de que todas as atividades que não produzem mercadoria (valor), são consideradas modalidades subalternas. Assim, criaram-se as bases que subjugam o trabalho doméstico à ideia de atividade improdutiva, que não gera riqueza e é visto como inferior perante as atividades de produção. O trabalho voltado para a reprodução foi tomado como uma atribuição feminina. A partir daí, uma série de qualidades são evocadas como naturalmente desenvolvidas nas mulheres, ao mesmo tempo em que promove a submersão e o apagamento dos processos históricos de construção social de sistemas de valorização e desvalorização; processos que sustentaram a divisão de trabalho de homens e mulheres e seu sistema hierárquico de atribuição de valor. Nesses processos, se consolidam a invisibilidade e a desvalorização do labor doméstico realizado pelas mulheres, mesmo quando ele passa a ser remunerado e é incorporado ao mercado de trabalho^5^.

O contexto, a vivência e a divisão do trabalho doméstico historicamente foram desiguais entre os sexos^5^. Apesar do movimento emancipatório das mulheres e da sua inserção no mercado de trabalho ter alcançado significativos avanços, a atividade doméstica, especialmente a não remunerada, continua sendo tarefa pouco compartilhada com os homens^6^. As mulheres permanecem as principais responsáveis pela realização e manutenção do trabalho doméstico não remunerado e enfrentam sobrecarga por terem que conciliar demandas de sua profissão e os cuidados com a casa e a família^10,11^.

Apesar dos mecanismos que mantêm as condições de subordinação e invisibilidade social, o trabalho doméstico é crucial para a existência humana e é a base para as atividades de reprodução: a sociedade continua existindo porque nela se desenvolvem ações de cuidado. Além de gerar bem-estar, esse tipo de trabalho contempla um conjunto de atividades diárias realizadas em prol da sobrevivência dos seres humanos^12^, é executado por meio de ações voltadas ao planejamento, organização e limpeza da casa e por ações diretas de cuidado na execução de serviços pessoais a outros indivíduos^13^. Assim, é possível, do ponto de vista teórico, conceber o trabalho doméstico envolvendo duas dimensões: realização de tarefas específicas (limpar, cozinhar, lavar, passar, fazer compras, dentre outras) e atividades de cuidado (cuidar dos filhos, de idosos, pessoas doentes e incapacitadas). Essas dimensões específicas envolvem intensidade, investimento de tempo e níveis de demanda diferenciados. Embora não sejam equivalentes (em quantidade e intensidade de esforços), o limite que separa essas dimensões é tênue, já que podem ser executadas conjuntamente, pois há cuidado em cozinhar, lavar e passar para outrem^13^. Apesar disso, são duas dimensões que devem compor os indicadores de avaliação do trabalho doméstico, permitindo identificar as cargas laborais envolvidas nessas atividades e as fontes de exposição à saúde física e mental implicadas na sua execução.

O trabalho doméstico, assim como as demais atividades laborais existentes, pode ser a fonte de agravos à saúde mental e diversos fatores contribuem, direta e indiretamente, para o bem-estar e a saúde das pessoas, como a multiplicidade de papéis desempenhados, as elevadas cargas de trabalho doméstico^10^, a quantidade de tempo gasto nas tarefas domésticas^14,15^, os conflitos decorrentes das dificuldades em conciliar diferentes atividades de trabalho, família e tempo para saúde e lazer^16^, a menor percepção de apoio do parceiro ao realizar trabalho domestico^17,18^, a divisão desigual dos cuidados com os filhos^9^ e da casa^19^, a exposição ao estresse contínuo associado ao trabalho doméstico^20^ e às situações estressoras decorrente do desequilíbrio entre esforços e demandas e o baixo reconhecimento do trabalho doméstico^21,22^.

No âmbito internacional, existe certa disponibilidade de estudos que exploram a relação do trabalho doméstico não remunerado e o adoecimento mental entre homens e mulheres; no Brasil, contudo, essa lacuna do conhecimento ainda persiste. Na maior parte das vezes, os estudos brasileiros se limitam a focalizar as frequências do adoecimento mental entre as mulheres e os fatores associados e, muito raramente, focalizam aspectos relativos ao trabalho doméstico^23^. A análise comparativa da relação entre trabalho doméstico e saúde entre homens e mulheres também é escassa.

Considerando os aspectos acima descritos que caracterizam processos contínuos de subordinação e invisibilidade das atividades da esfera da reprodução, do papel atribuído às mulheres nessa ocupação, aliado ao baixo investimento das pesquisas brasileiras na análise dessa divisão sexual do trabalho e a saúde mental de homens e mulheres, este estudo avaliou aspectos do trabalho doméstico não remunerado e sua associação com transtornos mentais, explorando diferenciais de gênero. Na exploração dos dados proposta neste artigo, as duas dimensões do trabalho doméstico foram mensuradas (atividades domésticas e de cuidados com a família), assim como os estressores ocupacionais presentes nesse tipo de trabalho e o conflito trabalho-família-tempo para si. Em conjunto, a análise desses aspectos permite identificar a persistência dos modelos tradicionais de divisão sexual do trabalho doméstico não remunerado e os desfechos negativos na saúde mental mais relevantes entre as mulheres.

## MÉTODOS

Foram analisados dados transversais preliminares de 2.841 participantes da segunda onda da coorte prospectiva “Vigilância em saúde mental e trabalho: uma coorte da população de Feira de Santana-BA”, financiada pela Fundação de Amparo à Pesquisa do Estado da Bahia (FAPESB)/Secretaria da Saúde do Estado da Bahia (SESAB), em parceria com o Conselho Nacional de Desenvolvimento Científico e Tecnológico (CNPq) e Ministério da Saúde (MS), por meio do “Programa pesquisa para o SUS: gestão compartilhada em saúde (PPSUS)” (PPSUS/BA – FAPESB/SESAB/CNPQ/MS 028/2018).

A coorte incluiu indivíduos com 15 anos de idade ou mais, residentes em zonas urbanas do maior município do interior do nordeste brasileiro. A amostra é proveniente de amostragem estratificada (por subdistritos) com sorteio de conglomerados em dois estágios: setores censitários e ruas. Em cada estrato (subdistrito) foi realizada amostragem simples das unidades primárias de amostragem (setor censitário) e dentro de cada uma foi realizada amostragem probabilística proporcional ao número de domicílios por rua e média estimada de pessoas por domicílio, por faixa etária e sexo, com dados disponibilizados pelo Instituto Brasileiro de Geografia e Estatística (IBGE). A partir dessas estimativas, foi determinado o número de ruas a serem sorteadas, as quais foram selecionadas aleatoriamente. Foram incluídos para as entrevistas todos os domicílios das ruas sorteadas, nos quais foram elegíveis os indivíduos residentes com 15 anos de idade ou mais. Com o intuito de reduzir as perdas, foram realizadas até três visitas aos domicílios para entrevistar cada morador elegível. As visitas foram realizadas em todos os dias da semana, no turno diurno, incluindo os sábados.

Os dados foram coletados entre dezembro de 2018 e novembro de 2019, por meio de entrevistas face-a-face realizadas no próprio domicílio de forma individual. A equipe foi orientada a realizar a entrevista, na medida do possível, de modo a favorecer a privacidade do(a) entrevistado(a), em função das questões subjetivas focalizadas na entrevista. A equipe de pesquisa foi previamente treinada em oficinas e reuniões. Para o trabalho de campo, foi elaborado um Manual de Condutas e Procedimentos para fins de padronização da coleta de dados.

Como instrumentos de coleta de dados, foram utilizados: a) Ficha de identificação domiciliar, para registrar a localização geográfica (subdistrito, setor censitário e endereço da residência), cadastrar os moradores do domicílio (elegíveis ou não) e as condições de moradia; b) Questionário individual multidimensional, estruturado em dez blocos temáticos, dos quais foram retirados os dados analisados neste estudo (características sociodemográficas, ocupacionais, do trabalho doméstico e adoecimento mental, estratificadas por sexo).

Além das características gerais do trabalho doméstico não remunerado, foram incluídas a escala de conflito trabalho-família-tempo para si (CTFT) e de desequilíbrio esforço-recompensa no trabalho doméstico (DER-doméstico).

O conflito trabalho-família-tempo avalia a impossibilidade ou dificuldade de conciliar os papeis assumidos nas diferentes dimensões da vida (trabalho, família e tempo para si)^27^. A abordagem do Conflito Trabalho-Família, tendo gênero como categoria analítica, se articula com a problemática da divisão sexual do trabalho na interrelação entre trabalho remunerado e vida doméstica como potenciais geradores de estresse e, consequentemente, de efeitos sobre a saúde de mulheres e homens inseridos no trabalho profissional^16^. Neste estudo, o indicador foi construído a partir da soma da pontuação de duas questões que avaliaram o conflito na direção do trabalho interferindo na família e uma que avaliou a dimensão do tempo para cuidado pessoal e lazer. Esses itens foram respondidos em uma escala tipo Likert, com pontuação de zero a quatro, validados para uso no Brasil^16^. A pontuação total foi utilizada para classificar o conflito trabalho-família-tempo para si em baixo, moderado ou alto, de acordo com os tercis.

A escala DER-doméstico avalia estressores psicossociais decorrentes do trabalho doméstico e familiar não remunerado, a partir da interação de três dimensões: esforço (aspectos extrínsecos, relacionados às obrigações e às demandas), recompensa (aspectos extrínsecos, relacionados ao reconhecimento, à valorização do trabalho, e ao sentimento de justiça) e comprometimento excessivo (componente intrínseco, relacionado ao envolvimento pessoal com suas atribuições da casa e da família)^22^. A hipótese desse modelo assume que o desequilíbrio entre os esforços empreendidos e as recompensas obtidas com o trabalho doméstico e familiar produz efeitos negativos sobre a saúde e a vida^22^. A avaliação desse item foi realizada por uma escala traduzida e validada no Brasil^21^, contendo 22 itens com opções de resposta em escala do tipo Likert (com pontuação de um a quatro). Neste estudo, para a composição do indicador de desequilíbrio, foram utilizadas a soma das pontuações das dimensões: esforço (sete itens) e recompensa (dez itens). O escore de desequilíbrio foi estimado pela equação: (Esforço)/(Recompensa x fator de correção), onde valores maiores que um, posteriormente, foram classificados em DER-doméstico baixo, moderado e alto.

Os transtornos mentais foram avaliados por meio de instrumentos de pesquisa de ampla utilização em todo o mundo e validados no Brasil, apresentando bom desempenho na avaliação do que se propõem a mensurar. Os Transtornos Mentais Comuns (TMC) foram avaliados pelo *Self-Reporting Questionnaire* (SRQ-20); Transtorno de Ansiedade Generalizada (TAG), avaliado pelo módulo de ansiedade do *Patient Health Questionnaire* (PHQ); e Depressão, avaliada pelo PHQ-9.

O SRQ-20 é um instrumento multidimensional, validado no Brasil^28^, constituído por 20 itens com opções de respostas dicotômicas (0-não e 1-sim), que avaliam sintomas de morbidade não psicótica (insônia, fadiga, irritabilidade, esquecimento, dificuldade de concentração e queixas somáticas). A suspeição de transtornos mentais comuns foi definida pela presença de sete ou mais respostas positivas para mulheres e cinco ou mais para os homens^29^.

O PHQ^30^ é um instrumento de avaliação de transtornos mentais elaborado com base nos critérios do Manual Diagnóstico e Estatístico de Transtornos Mentais (DSM), adaptado transculturalmente para uso no Brasil^31^. O PHQ é organizado em módulos para mensuração de agravos psíquicos específicos. Para este estudo, foram utilizados os módulos de transtorno de ansiedade generalizada (TAG) e depressão. O módulo de TAG contém sete itens para avaliar a frequência com que a pessoa se sentiu incomodada por problemas relacionados à ansiedade nas últimas quatro semanas. As opções de resposta estão expressas em escala do tipo Likert, variando de (0) “nenhuma” a (2) “mais que a metade dos dias”. A presença de TAG é definida por quatro ou mais respostas “mais que a metade dos dias” e, obrigatoriamente, entre elas, resposta positiva para o item “sentir-se nervoso, ansioso, tenso ou muito preocupado”^32^. A depressão foi avaliada pelo PHQ-9, versão validada para uso na população brasileira^33^. Esse módulo avalia a presença de sintomas, nas últimas duas semanas, por meio de nove itens com categorias de resposta do tipo Likert de quatro pontos: nenhum dia (0), menos de uma semana (1), uma semana ou mais (2) e quase todos os dias (3). O somatório das respostas é utilizado para calcular o escore total, sendo o ponto de corte ≥ 10 recomendado para o diagnóstico de depressão^32^.

O estudo buscou responder as seguintes perguntas: persiste a diferença na responsabilização do trabalho doméstico entre os sexos? O conflito trabalho-família-tempo para si e desequilíbrio esforço-recompensa no trabalho doméstico e familiar estão associados a transtornos mentais entre os homens e entre as mulheres de forma desigual?

A análise conduzida teve caráter exploratório e, inicialmente, foram avaliadas as características do trabalho doméstico não remunerado. Em seguida, estimadas prevalências, razões de prevalência e respectivos intervalos de confiança de 95%. As análises foram processadas pelos programas *Statistical software for data science* (Stata), versão 15, e *Statistical Package for the Social* Science (SPSS), versão 24.

O estudo atendeu aos critérios éticos estabelecidos para pesquisa envolvendo seres humanos e possui aprovação do Comitê de Ética em Pesquisa da Universidade Estadual de Feira de Santana (CAAE: 74792617.4.0000.0053).

## RESULTADOS

A amostra foi composta por 2.841 pessoas, a maioria (66,7%) mulheres. Para ambos os sexos predominou: idade entre 31 e 59 anos (homens: 42,8%; mulheres: 46,8%); não ter companheiro(a) (homens: 50,1%; mulheres: 56,4%); ensino médio como escolaridade (homens: 47,2%; mulheres: 45,5%); negras(os) (homens: 83%; mulheres: 81%); renda mensal até um salário-mínimo (homens: 47,2%; mulheres: 59,5%). Mais da metade dos homens tinha trabalho remunerado (68,1%), enquanto apenas 47,2% das mulheres exerciam atividade laboral remunerada. Entre os(as) trabalhadores(as) remunerados(as) predominou a informalidade (homens: 74,3%; mulheres: 75,4%) e jornada de trabalho de até cinco dias (homens: 50,8%; mulheres: 53,1%). A carga horária de trabalho semanal de até 30 horas foi observada nas respostas de 34,7% dos homens e de 48,3% das mulheres; a jornada integral (mais de 30 horas semanais) foi referida por 65,3% dos homens e 56,2% das mulheres (Tabela 1).

**Tabela 1:** Características sociodemográficas, ocupacionais e do trabalho doméstico não remunerado, segundo o gênero. Feira de Santana, Bahia, 2019, (n = 2.841).

Variáveis (n)	Homens		Mulheres		p
	
	n	%	n	%	
**Características sociodemográficas**					
Faixa etária (anos) (2.832)					
15–30	304	32,2	455	24,1	< 0,001
31–59	404	42,8	883	46,8	
≥ 60	236	25,0	550	29,1	
Situação conjugal (2.796)					
Com companheiro(a)	461	49,9	816	43,6	0,002
Sem companheiro(a)	463	50,1	1056	56,4	
Escolaridade (2.796)					
Ensino fundamental	371	40,1	718	38,4	0,060
Ensino médio	437	47,2	850	45,5	
Ensino superior	118	12,7	302	16,1	
Raça/cor (2.690)					
Não negros(as)	150	17,0	343	19,0	0,216
Negros(as)	732	83,0	1465	81,0	
Renda mensal (salário-mínimo^a^) (1.438)					
≤ 1	244	47,2	548	59,5	< 0,001
1–2	179	34,6	268	29,1	
> 2	94	18,2	105	11,4	
Com quem reside (2.796)					
Familiares/amigos(as)	823	88,6	1709	91,5	0,012
Sozinho	106	11,4	158	8,5	
**Características ocupacionais**					
Situação de trabalho remunerado (1.809)^b^					
Trabalha	448	68,1	543	47,2	< 0,001
Não trabalha	210	31,9	608	52,8	
Vínculo de trabalho (1.017)^c^					
Formal	117	25,7	138	24,6	0,698
Informal	339	74,3	423	75,4	
Dias de trabalho (por semana) (959)					
≤ 5	222	50,8	277	53,1	0,485
6–7	215	49,2	245	46,9	
Horas de trabalho (por semana) (927)					
≤ 30	144	34,7	224	43,8	0,005
> 30	271	65,3	288	56,2	
**Trabalho doméstico**					
Faz atividade(s) doméstica(s) (n = 2.483)					
Não	217	28,7	83	4,8	< 0,001
Sim	539	71,3	1644	95,2	
Lavar roupa (2.631)					
Não	471	58,8	283	15,5	< 0,001
Sim	330	41,2	1547	84,5	
Passar roupa (2.618)					
Não	633	79,1	884	48,6	< 0,001
Sim	167	20,9	934	51,4	
Limpar a casa (2.637)					
Não	325	40,5	153	8,3	< 0,001
Sim	478	59,5	1681	91,7	
Cozinhar (2.632)					
Não	447	55,9	236	12,9	< 0,001
Sim	353	44,1	1596	87,1	
Fazer feira/compra de supermercado (2.625)					
Não	333	41,7	391	21,4	< 0,001
Sim	465	58,3	1436	78,6	
Faz pequenos consertos (2.619)					
Não	361	45,1	1290	70,9	< 0,001
Sim	439	54,9	529	29,1	
Cuidar de crianças ≤ 5 anos (2.582)					
Não	673	86,2	1269	70,5	< 0,001
Sim	108	13,8	532	29,5	
Cuidar de idosos, doentes ou com necessidades especiais (2.588)					
Não	710	90,6	1478	81,9	< 0,001
Sim	74	9,4	326	18,1	
Recebe apoio no trabalho doméstico^d^ (2.406)					
Sim	322	48,8	864	49,5	0,760
Não	338	51,2	882	50,5	
Tem empregada(o) doméstica(o) (2.760)					
Sim	67	7,3	109	5,9	0,149
Não	847	92,7	1737	94,1	
CTFT^e^ (1.038)					
Baixo	139	39,0	196	28,8	0,003
Moderado	97	27,3	213	31,2	
Alto	120	33,7	273	40,0	
DER- Doméstico^e^ (2.071)					
Baixo	228	45,8	447	28,4	< 0,001
Moderado	102	20,5	588	37,4	
Alto	168	33,7	538	34,2	

^a^ Salário-mínimo em 2019: R$ 998,00.

^b^ Considerou a condição de trabalho atual do indivíduo, excluindo estudantes, aposentados(as), pensionistas, donas de casa ou quem vivia de renda.

^c^ Este N considerou vínculo de trabalho sobre empregos no último ano (independente de trabalho atual).

^d^ O apoio na realização das atividades domésticas, segundo quem o fornecia, teve a seguinte distribuição (possibilidade de múltiplas respostas): empregado(a) doméstica: 5,8%; outros homens: 18,8%; marido/companheiro: 23,1%; esposa/companheira: 21,7%; outras mulheres 89,4%.

^e^ CTFT (conflito trabalho-família-tempo para si) foi respondido por pessoas que tiveram trabalho remunerado no último ano e faziam atividades domésticas e DER-doméstico (desequilíbrio esforço-recompensa-doméstico e familiar) foi respondido por pessoas que afirmaram realizar trabalho doméstico não remunerado.

A pesquisa apontou que as mulheres são as principais responsáveis pela realização de atividades domésticas, assumidas por 95,2% das entrevistadas, enquanto 71,3% dos homens realizavam esse tipo de tarefa. Esse retrato se modifica quando se observam os dados de pequenos consertos que foram referidos por 54,9% dos homens e 29,1% das mulheres. Dentre as atividades domésticas às quais as mulheres eram as maiores responsáveis estavam: lavar roupa (homens: 71,3%; mulheres: 95,2%); passar roupa (homens: 20,9%; mulheres: 51,4%); limpar a casa (homens: 59,5%; mulheres: 91,7%); cozinhar (homens: 44,1%; mulheres: 87,1%); fazer compras (homens: 58,3%; mulheres: 78,6%).

A atribuição do trabalho doméstico que envolve as atividades de cuidado com a família também recaiu principalmente sobre as mulheres, dentre elas cuidar de crianças até cinco anos (homens: 13,8%; mulheres: 29,5%) e cuidar de idosos, doentes ou pessoas com necessidades especiais (homens: 13,8%; mulheres: 29,5%). Ter apoio na realização do trabalho doméstico foi referido por 51,2% dos homens e 50,5% das mulheres. Também foi avaliado quem ofereceu o apoio – empregada(o) doméstica(o), cônjuge, outras mulheres (mãe/irmã/filha/ vizinha) ou outros homens (pai/irmão/filho/vizinho): o maior percentual foi o apoio recebido de outra mulher, totalizando 89,4%. Apoio de empregada(o) doméstica(o) foi referido por 7,3% dos homens e 5,9% das mulheres.

Entre as mulheres predominou situação de elevado conflito trabalho-família-tempo para si (40,0%), seguido de moderado (28,8%); entre os homens o maior percentual foi de baixo conflito (39,0%). Para o desequilíbrio esforço-recompensa com o trabalho doméstico, predominou, entre as mulheres, o nível moderado (37,4%), seguido de alto (34,2%); entre os homens prevaleceu o nível baixo (45,8%) (Tabela 1).

Na população estudada, as prevalências dos transtornos mentais foram mais expressivas entre as mulheres: a prevalência de transtornos mentais comuns foi de 25,5% contra 21,7% dos homens; de transtorno de ansiedade generalizada foi 6,8% entre as mulheres e de 2,4% entre os homens; e a depressão foi constatada em 10,9% das mulheres e 5,5% dos homens (Dados não apresentados em tabelas).

O alto nível de conflito trabalho-família-tempo foi significativamente associado aos transtornos mentais comuns tanto em homens (RP = 1,69; IC95% 1,12–2,55) quanto em mulheres (RP = 1,86; IC95% 1,31–2,66), e a depressão apenas entre as mulheres (RP = 1,99; IC95% 1,05–3,75) (Tabela 2).

**Tabela 2:** Prevalência (%), razões de prevalência e intervalos de confiança de 95% para associação de transtornos mentais, segundo níveis de desequilíbrio esforço-recompensa e conflito trabalho-família e segundo gênero. Feira de Santana, Bahia, 2019.

Variáveis	Homens				Mulheres			
	
	P	IC95%	RP	IC95%	P	IC95%	RP	IC95%
**Transtornos Mentais Comuns**^a^								
Conflito trabalho-família								
Baixo	20,9	14,4–28,8	1,00	–	17,3	12,2–23,4	1,00	–
Moderado	24,0	15,8–33,7	1,15	0,71–1,86	24,6	18,9–31,0	1,43	0,97–2,11
Alto	35,3	26,7–44,8	1,69	1,12–2,55	32,2	26,6–38,2	1,86	1,31–2,66
DER-doméstico								
Baixo	24,7	19,1–30,9	1,00	–	16,7	13,3–20,5	1,00	–
Moderado	34,0	24,8–44,1	1,38	0,97–1,97	26,3	22,7–30,1	1,57	1,23–2,02
Alto	28,4	21,5–36,0	1,15	0,82–1,61	30,6	26,6–34,7	1,83	1,43–2,34
**Transtorno de Ansiedade Generalizada**^b^								
Conflito trabalho-família								
Baixo	1,4	0,2–5,1	1,00	–	4,7	2,1–8,6	1,00	–
Moderado	3,1	0,6–8,8	2,13	0,36–12,6	6,2	3,3–10,3	1,32	0,58–3,02
Alto	3,4	0,9–8,4	2,32	0,43–12,5	8,2	5,2–12,2	1,76	0,83–3,74
DER-doméstico								
Baixo	2,2	0,7–5,1	1,00	–	3,4	1,9–5,6	1,00	–
Moderado	2,0	0,2–6,9	0,89	0,17–4,46	7,8	5,7–10,3	2,30	1,30–4,06
Alto	5,4	2,5–10,0	2,42	0,83–7,12	8,3	6,1–11,0	2,44	1,37–4,32
**Depressão**^c^								
Conflito trabalho-família								
Baixo	3,6	1,1–8,3	1,00	–	6,2	3,2–10,6	1,00	–
Moderado	3,1	0,6–8,7	0,85	0,21–3,47	11,9	7,8–17,0	1,92	0,99–3,71
Alto	9,3	4,7–16,1	2,55	0,91–7,15	12,4	8,6–16,9	1,99	1,05–3,75
DER-doméstico								
Baixo	4,8	2,4–8,4	1,00	–	4,7	3,0–7,2	1,00	–
Moderado	7,8	3,5–14,9	1,63	0,67–3,92	10,7	8,3–13,5	2,26	1,40–3,65
Alto	10,8	6,5–16,5	2,23	1,08–4,61	13,9	11,1–13,9	2,93	1,83–4,67

P: prevalencia; RP: razão de prevalência.

^a^ Avaliado pelo SRQ-20.

^b^ Avaliado pelo módulo de ansiedade do PHQ.

^c^ Avaliado pelo PHQ9.

Entre as mulheres, os níveis de desequilíbrio esforço-recompensa moderado e alto se associaram significantemente aos transtornos mentais comuns (RP = 1,57; IC95% 1,23–2,02; e RP = 1,83; IC95% 1,43–2,34 respectivamente), ao transtorno de ansiedade generalizada (RP = 2,30; IC95% 1,30–4,06; e RP = 2,44; IC95% 1,37–4,32 respectivamente) e à depressão (RP = 2,26; IC95% 1,40–3,65; e RP = 2,93; IC95% 1,83–4,67, respectivamente). Entre os homens, o DER-doméstico alto associou-se significativamente apenas à depressão (RP = 2,23; IC95% 1,08–4,61).

## DISCUSSÃO

Os resultados deste estudo evidenciaram a persistência da divisão sexual do trabalho, tanto nos aspectos relacionados às atividades remuneradas quanto não remuneradas, com maiores desvantagens para as mulheres. As proporções de exclusão do mercado de trabalho, de menor renda mensal, de menor remuneração salarial, trabalho remunerado informal e jornada de trabalho parcial também foram maiores entre as mulheres do que as proporções encontradas entre os homens. Tais aspectos, que evidenciam diferenças de gênero na participação no mercado de trabalho e no acesso aos seus benefícios, podem influenciar no aumento do tempo e intensidade na participação das atividades domésticas e de cuidado não remunerados^5,8^. O menor acesso à renda torna menos viável o apoio profissional na realização dessas atividades (seja por meio de creches, contratação de trabalhadora/o doméstica/o remunerada/o). Paralelamente, uma menor participação no mercado laboral favorece uma maior responsabilização pelo trabalho doméstico, o que, por sua vez, limita as possibilidades de qualificação e formação profissional. Assim, a histórica maior responsabilização, torna o acesso das mulheres ao mercado de trabalho mais difícil^7^, promovendo e alimentando os ciclos de exclusão e perpetuando os mecanismos de produção e reprodução das desigualdades de gênero.

A realização das atividades domésticas foi relatada pela maioria dos participantes, contudo, as mulheres eram as principais responsáveis, exceto para pequenos consertos. Estudos anteriores identificaram pouca participação dos homens em atividades domésticas, classificando-a como ocasional, flexível e limitada aos pequenos consertos^1^. Atividades como cuidar dos filhos, limpar, lavar roupas, arrumar e cozinhar seguem sendo executadas majoritariamente pelas mulheres^10^. Assim, observou-se continuidade das concepções estruturantes patriarcais que remetem às mulheres a responsabilidade das atividades domésticas não remuneradas, independentemente da inserção no mercado de trabalho.

Os resultados deste estudo indicaram comprometimento significativo da saúde mental das mulheres, corroborando com o encontrado na literatura, que aponta maior prevalência de transtornos mentais entre as mulheres, sendo os transtornos mais frequentes aqueles relacionados aos sintomas de ansiedade, humor depressivo, insônia, anorexia nervosa e sintomas psicofisiológicos^10^. Enquanto, entre os homens destacam-se os distúrbios de conduta, como uso de drogas e abuso de álcool^10^.

Estudos internacionais consistentemente já apontavam associação entre características do trabalho doméstico não remunerado e saúde física e mental^9,14,15,17^. Os estudos brasileiros também evidenciaram maior adoecimento mental entre as mulheres^10,21^. Quando ambos os sexos foram avaliados, ficou evidente o maior efeito negativo sobre a saúde mental das mulheres^16^, tal como os estudos internacionais.

Os dados comprovam que as mulheres não apenas estavam mais envolvidas e eram as principais responsáveis pelo trabalho doméstico como também vivenciavam percentuais mais elevados de conflitos nas diferentes dimensões da vida (trabalho-família-tempo para si) e eram mais afetadas pelos estressores do trabalho doméstico, experimentando situações de desequilíbrio esforço-recompensa. Assim, as dificuldades/impossibilidades de conciliar múltiplos papeis foram mais recorrentes entre as mulheres As associações entre esses aspectos e adoecimento mental foram consistentes para as mulheres, sugerindo que as diferenças na execução de trabalho doméstico não remunerado relacionam-se aos efeitos adversos mais proeminentes entre elas.

Estudo que avaliou associação entre conflito trabalho-família-tempo pessoal e ansiedade generalizada identificou maior proporção do conflito e prevalência de transtorno de ansiedade em mulheres, bem como maior força de associação entre as variáveis^16^.

A exposição ao desequilíbrio esforço-recompensa impactou negativamente sobre a saúde mental. A desvalorização das atividades domésticas, a maior responsabilidade, o volume de tarefas desempenhadas, bem como o sentimento de injustiça pela execução do trabalho, desequilibra a relação entre esforços necessários para desenvolver tais atividades e as recompensas/reconhecimentos recebidos, gerando estressores que se associam ao adoecimento físico e mental, especialmente das mulheres^21^.

Este é um estudo exploratório, desenvolvido com o objetivo de analisar características do trabalho doméstico não remunerado, focalizando a sua distribuição e avaliando sua relação com os transtornos mentais mais frequentes em populações urbanas (transtornos mentais comuns, depressão e transtornos de ansiedade generalizada). Assim, os resultados obtidos devem ser analisados com cautela, uma vez que correspondem a uma aproximação inicial das análises entre esses eventos, sem considerar ajustes ou interações entre as variáveis. Apesar dessa limitação, o estudo investigou uma amostra representativa da população-alvo da maior cidade do interior do nordeste brasileiro, incluindo amostra de homens e mulheres. Adicionalmente, analisou uma dimensão da vida relacionada à reprodução, que raramente é focalizada ou, quando analisada, em geral restringe-se à análise das mulheres.

A avaliação de estressores no trabalho doméstico no contexto brasileiro, a partir de uma amostra de população geral, utilizou uma ferramenta de produção de dados potente, até então de uso restrito. A análise nessa direção evidenciou a persistência de desvantagens para as mulheres não somente na atribuição de responsabilidades pela casa e família, mas também identificou a produção de estressores associados a essas atribuições. O instrumento (DER-doméstico), adaptado de um dos modelos de estressores ocupacionais mais utilizados no mundo do trabalho remunerado^34^, traz novas possibilidades para melhor identificar exposições nesse tipo de trabalho, ainda oculto e desvalorizado. Permite, assim, vislumbrar análise de indicadores úteis na identificação de situações desfavoráveis, podendo orientar ações, políticas públicas e alternativas para redução de desigualdades sociais em saúde relacionadas ao gênero.

A persistência de desigualdades na divisão do trabalho doméstico não remunerado segundo gênero e maior repercussão na saúde mental feminina foram observadas. É provável que a pandemia de covid-19 tenha intensificado essas desigualdades, uma vez que a rede de apoio (escolas, creches, parentes, babás) tornou-se indisponível e as demandas por limpeza ampliaram-se nesse contexto. Adicionalmente, o trabalho remoto trouxe as atividades remuneradas para casa, acumulando, em um mesmo espaço, sem limites temporais claros, o mundo do trabalho e de cuidados com a família. A sobreposição de exigências e demandas na casa podem potencializar os conflitos e os estressores ocupacionais. A discussão da relação entre gênero/trabalho doméstico/saúde mental continua oculta, mas os resultados obtidos evidenciaram indicadores importantes de desigualdades que precisam ser enfrentados como problemas de saúde pública. É preciso desconstruir os muros que enclausuraram o trabalho doméstico no âmbito privado e analisá-lo como determinante de adoecimento. Estudos comparativos pré e pós pandemia podem dimensionar novas consequências dessa equação e contribuir na elaboração de políticas públicas para reduzir as desigualdades identificadas.
